# Supplementary material for: Noradrenergic arousal after encoding reverses the course of systems consolidation in humans
Source: Nat Commun. 2021 Oct 18;12:6054. doi: 10.1038/s41467-021-26250-7 (PMC8523710; doi:10.1038/s41467-021-26250-7)
Supplement: Supplementary file 1 — Supplementary Information [file 41467_2021_26250_MOESM1_ESM.pdf]

SUPPLEMENTARY INFORMATION

Noradrenergic arousal after encoding reverses the course of systems consolidation in humans

Valentina Krenz[1], Tobias Sommer[2], Arjen Alink[2], Benno Roozendaal[3,4], and Lars Schwabe[1*]

[1]Department of Cognitive Psychology, Institute of Psychology, Universität Hamburg, Von-Melle-Park 5, 20146 Hamburg, Germany
[2]University Medical Centre Hamburg-Eppendorf, Department of Systems Neuroscience, Martinistraße 52, 20246, Hamburg, Germany
[3]Department of Cognitive Neuroscience, Radboud university medical center, 6500 HB Nijmegen, The Netherlands
[4]Donders Institute for Brain, Cognition and Behaviour, Radboud University, Kapittelweg 29, 6525 EN Nijmegen, The Netherlands

* Lars Schwabe, Department of Cognitive Psychology, Institute of Psychology, University of Hamburg, Von-Melle-Park 5, 20146 Hamburg, Germany. Phone: +49-40-428385950.
Email: Lars.schwabe@uni-hamburg.de

Supplementary methods

Stimulus material. We used 120 pictures of scenes and objects as stimulus material (as well as 120 additional stimuli which will be analyzed elsewhere), taken from the International Affective Picture System[1] and open internet platforms. Half of the pictures contained emotionally negative scenes or objects while the other half contained neutral contents. At the end of the experiment, participants rated all pictures with respect to picture valence and arousal on a scale from 0 ("very negative"/"not arousing") to 10 ("very positive"/"very arousing"). As expected, negative pictures were rated as significantly more negative (M=2.52, SD=0.69) than neutral pictures (M=5.62, SD = 0.81; paired *t*-test: t_{103}=24.41, p<0.001, d=2.39). Furthermore, negative pictures (M=5.70, SD=1.30) were rated as more emotionally arousing than neutral pictures (M=2.74, SD=1.45; paired *t*-test: t_{103}=-23.03, p<0.001, d=2.26).

Supplementary results

Control Variables. The four experimental groups did not differ in subjective chronic stress levels, depressive mood, state or trait anxiety, sleep quality or quantity in the time between encoding and recognition testing (all F<2.53, all p>0.061, see supplementary table 1).

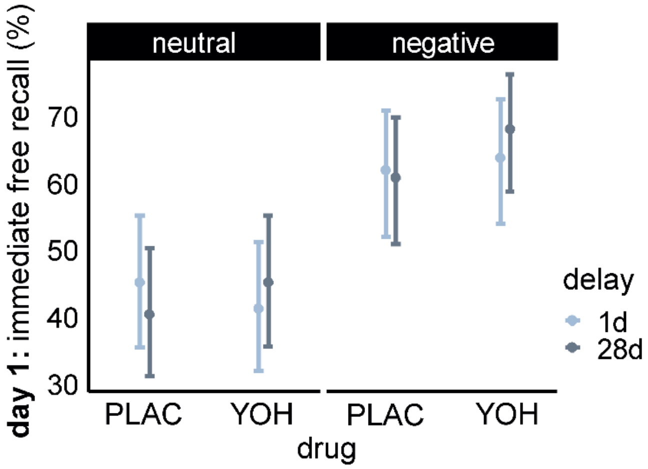

Supplementary figure 1. Predicted probability to remember items in the free recall task. Emotionally negative items were remembered significantly more likely than emotionally neutral ones (main effect emotion: β=1.98, z=3.1, p=0.001, generalized linear mixed model; n=104 participants). No differences were found between groups in this task indicating that yohimbine (YOH vs. placebo (PLAC)) did not influence memory during encoding. Bars represent predicted means with 95%-CIs. Source data are provided as Source Data file.

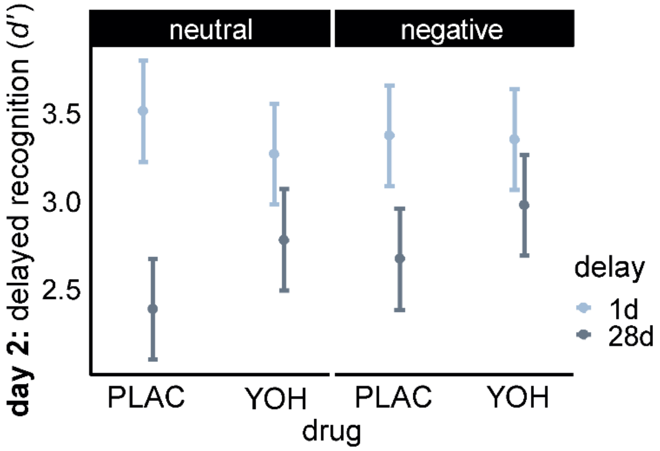

Supplementary figure 2. Predicted memory performance during delayed recognition testing. The time-dependent decrease in memory performance from the 1d- to the 28d-delayed test (β=-1.12, 95%-*CI*[-1.12,-0.72], $t_{125.82}$=- 5.43, p<0.001, linear mixed model (LMM); n=104 participants) was significantly lower for emotionally negative than neutral items (emotion×delay: β=0.42, 95%-*CI*[0.14, 0.70], t_{100}=2.98, p=0.003, LMM). Most importantly, the memory decline with increasing delay was weaker in the yohimbine (YOH) group than in the placebo (PLAC) group (drug×delay: β=0.64, 95%-*CI*[0.06, 1.21], $t_{125.816}$=2.18, p=0.029, LMM) irrespective of the emotionality of the encoded stimuli (drug×delay×emotion: β=-0.31, p=0.124, LMM). Bars represent predicted means with 95%-CIs. Source data are provided as Source Data file.

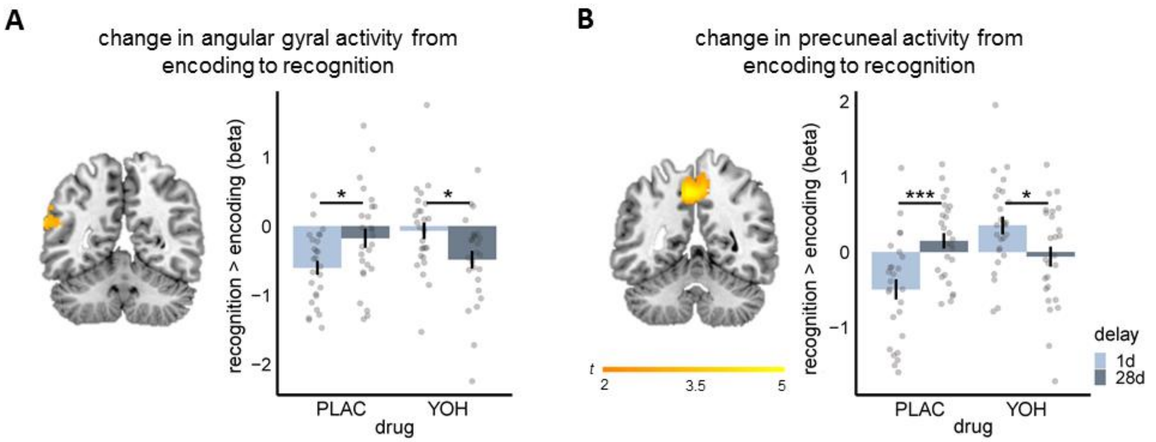

Supplementary figure 3. Noradrenergic stimulation decreases contribution for exploratory posterior regions over time. A While there was a significant increase in angular gyral activity from encoding to memory testing at the 28d vs. 1d delayed test in the placebo (PLAC) group ($t_{45.88}$=-2.51, p=0.016, d=0.70, two-tailed Welch's t-test), there was even a significant decrease in angular gyral activity from encoding to retrieval with increasing retention delay in the yohimbine (YOH) group (drug×delay: SVC peak-level: x=-62, y=-54, z=22, t=3.75, p_{corr}(FWE)=0.020, k=96; $t_{49.82}$=2.41, p=0.020, d=0.67, mixed ANOVA). **B** As in our exploratory whole-brain analysis, focusing on the precuneus as ROI revealed an interaction of drug×delay (SVC peak-level: x=-4, y=-50, z=46, t=5.21, p_{corr}(FWE)<0.001, k=877, mixed ANOVA) with a significant increase from 1d to 28d delayed test in the PLAC group ($t_{46.76}$=-3.82, p<0.001, d=1.06, two-tailed Welch's t-test), but a significant decrease in precuneal activity for the YOH group ($t_{49.30}$=2.34, p=0.02, d=0.65, two-tailed Welch's t-test).

All n=104 participants. Bonferroni Correction was applied for number of regions of interest in each analysis. Visualizations show t-maps for the interesting contrasts superimposed on sagittal sections of T1-weighted template images and beta-values for the significant cluster. Bars represent mean ± SEM. Source data are provided as Source Data file. ^{+}p<0.100; $^{**}p$<0.010; $^{***}p$<0.001

Supplementary table 1. Control variables.

Variable	PLAC		YOH	
	1d	28d	1d	28d
depressive mood (BDI-II)	4.23 (0.72)	3.77 (0.74)	6.69 (0.92)	4.81 (0.83)
state anxiety (STAI-S)	32.92 (0.67)	34.77 (1.43)	35.62 (1.25)	34.19 (1.16)
trait anxiety (STAI-T)	33.19 (1.16)	32.77 (1.44)	33.31 (1.25)	34.96 (1.27)
subjective chronic stress (TICS)	10.88 (1.06)	12.73 (1.21)	15.35 (1.53)	14.23 (1.57)
sleep quality (PSQI)				
global score (last 28d)	4.23 (0.39)	4.92 (0.53)	4.42 (0.43)	4.68 (0.53)
sleep quality (last 24h)	1.69 (0.12)	1.92 (0.12)	2.04 (0.20)	1.96 (0.15)
sleep latency (last 24h)	7.44 (0.22)	7.21 (0.26)	7.54 (0.33)	7.39 (0.24)

Data represents mean (SEM). Source data are provided as Source Data file.

Supplementary references

1. Lang, P. J., Bradley, M. M. & Cuthbert, B. N. *The International Affective Picture System (IAPS): Technical manual and affective ratings*. (NIMH Center for the Study of Emotion and Attention, Gainesville, 1997).
