# Supplementary material for: Noradrenergic arousal after encoding reverses the course of systems consolidation in humans
Source: Nat Commun. 2021 Oct 18;12:6054. doi: 10.1038/s41467-021-26250-7 (PMC8523710; doi:10.1038/s41467-021-26250-7)
Supplement: Supplementary file 3 — Reporting Summary [file 41467_2021_26250_MOESM3_ESM.pdf]

nature research

Corresponding author(s): Lars Schwabe

Last updated by author(s): Sep 1, 2021

Reporting Summary

Nature Research wishes to improve the reproducibility of the work that we publish. This form provides structure for consistency and transparency in reporting. For further information on Nature Research policies, see our Editorial Policies and the Editorial Policy Checklist.

Statistics

For all statistical analyses, confirm that the following items are present in the figure legend, table legend, main text, or Methods section.

n/a	Confirmed	
☐	☒	The exact sample size (*n*) for each experimental group/condition, given as a discrete number and unit of measurement
☐	☒	A statement on whether measurements were taken from distinct samples or whether the same sample was measured repeatedly
☐	☒	The statistical test(s) used AND whether they are one- or two-sided<br>*Only common tests should be described solely by name; describe more complex techniques in the Methods section.*
☐	☒	A description of all covariates tested
☐	☒	A description of any assumptions or corrections, such as tests of normality and adjustment for multiple comparisons
☐	☒	A full description of the statistical parameters including central tendency (e.g. means) or other basic estimates (e.g. regression coefficient) AND variation (e.g. standard deviation) or associated estimates of uncertainty (e.g. confidence intervals)
☐	☒	For null hypothesis testing, the test statistic (e.g. *F*, *t*, *r*) with confidence intervals, effect sizes, degrees of freedom and *P* value noted<br>*Give P values as exact values whenever suitable.*
☒	☐	For Bayesian analysis, information on the choice of priors and Markov chain Monte Carlo settings
☒	☐	For hierarchical and complex designs, identification of the appropriate level for tests and full reporting of outcomes
☐	☒	Estimates of effect sizes (e.g. Cohen's *d*, Pearson's *r*), indicating how they were calculated

Our web collection on statistics for biologists contains articles on many of the points above.

Software and code

Policy information about availability of computer code

Data collection	Experiments were implemented using MATLAB (www.mathworks.com) with the Psychophysics Toolbox 3 extensions. MRI data were acquired using a 3T Prisma Scanner (Siemens, Germany) with a 64-channel head coil
Data analysis	Behavioral and autonomic data analyses were performed with R version 4.0.2 (https://www.r-project.org/)<br>Preprocessing and analysis of the fMRI data was performed using SPM12 (Wellcome Trust Centre for Neuroimaging, London, UK) in MATLAB 2016b. Multivariate analysis was applied on the firstlevel using custom scripts in MATLAB 2016b and further analyzed using SPM12. The resulting parameter estimates were extracted using the MarsBar Toolbox (http://www.mrc-cbu.cam.ac.uk/Imaging/marsbar.html) for data visualization, to apply post-hoc t-tests and correlate neural activity estimates with memory performance.<br>Custom code used to analyze the data is available at Github: https://github.com/valentinakrenz/NorSysCons.

For manuscripts utilizing custom algorithms or software that are central to the research but not yet described in published literature, software must be made available to editors and reviewers. We strongly encourage code deposition in a community repository (e.g. GitHub). See the Nature Research guidelines for submitting code & software for further information.

Data

Policy information about availability of data

All manuscripts must include a data availability statement. This statement should provide the following information, where applicable:

- Accession codes, unique identifiers, or web links for publicly available datasets
- A list of figures that have associated raw data
- A description of any restrictions on data availability

Behavioral, autonomic, and fMRI data that support the findings of this study are available at Github: https://github.com/valentinakrenz/NorSysCons

Field-specific reporting

Please select the one below that is the best fit for your research. If you are not sure, read the appropriate sections before making your selection.

☐ Life sciences ☒ Behavioural & social sciences ☐ Ecological, evolutionary & environmental sciences

For a reference copy of the document with all sections, see nature.com/documents/nr-reporting-summary-flat.pdf

Behavioural & social sciences study design

All studies must disclose on these points even when the disclosure is negative.

Study description	Quantitative study in humans using fMRI, pharmacological manipulation, behavioral and autonomic measures.
Research sample	One-hundred-and-nine healthy, right-handed volunteers (55 males and 54 females, age: M=24.09 years, SD=3.92 years) participated in the study. Exclusion criteria were checked in a standardized interview and comprised a history of any psychiatric or neurological diseases, medication intake or drug abuse, kidney- and liver-related diseases, body-mass index below 19 or above 26 kg/m², diagnosed cardiovascular problems as well as any contraindications for MRI measurements or Yohimbine intake. The sample included both students and non-students, but psychology students were excluded to avoid subject-expectancy effects. This study was not representative of the general population.<br>The final sample size is in line with other fMRI studies on the effect of stress or stress mediators on memory and an a-priori power calculation with G*Power suggested that this sample size is sufficient to detect a medium-sized effect with a power of 0.80.
Sampling strategy	This final sample size is in line with other fMRI studies on the effect of stress or stress mediators on memory (e.g. Schwabe, Tegenthoff, Hoffken & Wolf 2012; Journal of Neuroscience) and an a-priori power calculation with G*Power 3.1 suggested that this sample size is sufficient to detect a medium-sized effect with a power of 0.80.
Data collection	During the free recall task, participants named all remembered items from the encoding task in as much detail as possible while the experimenter ticked off the remembered items from a list and an audiorecording was conducted. All other behavioral tasks were computerbased using MATLAB with the Psychophysics Toolbox extensions. Demographic and questionnaire data was assessed using electronic tablets. Autonomic data, i.e. blood pressure, was assessed using upper arm blood pressure monitors by OMRON Healthcare.<br>During testing, only the participant and the experimenter were present and both were blinded regarding the pharmacological condition (i.e. Yohimbine vs. Placebo).
Timing	Data collection took place between January 2019 and February 2020.
Data exclusions	Four participants had to be excluded from the analysis because of technical failure (n=1) or falling asleep during at least one of the MRI sessions (n=3).
Non-participation	One participant did not return for the second experimental day due to health related reasons.
Randomization	Participants were randomly assigned to the drug condition in a double-blinded manner, i.e. due to indistinguishable pills neither the experimenter nor the participant knew of the group assignment. Depending on their availability, participants completed experimental day 2 either 1 day or 28 days after the first experimental day. Therefore, assignment to the delay condition was pseudo-randomized.

Reporting for specific materials, systems and methods

We require information from authors about some types of materials, experimental systems and methods used in many studies. Here, indicate whether each material, system or method listed is relevant to your study. If you are not sure if a list item applies to your research, read the appropriate section before selecting a response.

Materials & experimental systems

n/a	Involved in the study	
☒	☐	Antibodies
☒	☐	Eukaryotic cell lines
☒	☐	Palaeontology and archaeology
☒	☐	Animals and other organisms
☐	☒	Human research participants
☒	☐	Clinical data
☒	☐	Dual use research of concern

Methods

n/a	Involved in the study	
☒	☐	ChIP-seq
☒	☐	Flow cytometry
☐	☒	MRI-based neuroimaging

Human research participants

Policy information about studies involving human research participants

Population characteristics	see above
Recruitment	This study used a convenience sample based on volunteers reacting to online job postings and flyers (for more information on see 'research sample').
Ethics oversight	The study protocol was approved by the ethics committee of the Medical Chamber Hamburg and was in accordance with the declaration of Helsinki. The instituttional review bpoard classified this study explicitly as a basic experimental study in humans.

Note that full information on the approval of the study protocol must also be provided in the manuscript.

Magnetic resonance imaging

Experimental design

Design type	Task-based fMRI with an event-related design.
Design specifications	The encoding task consisted of three consecutive runs of 7min each in which the same 60 stimuli were randomly presented, i.e. each stimulus was presented once in each run. In each trial, a picture was presented for 3s followed by a jittered fixation period for 4±1s. The recognition task was seperated into three consecutive runs with 80 trials each. In each trial, a picture was presented for 3s followed by a rating scale, which was presented until a response was given and for max. 3s. Between trials, a jittered fixation cross was presented for 4s±1s.
Behavioral performance measures	The sensitivity index d' was used as a bias-free indicator of memory performance. Missing responses during encoding and recognition were examined to control for the alertness during the fMRI.<br><br>Three participants resported falling asleep in the MRI and additionally showed a high amount of missing responses during at least one of the tasks and were therefore excluded.

Acquisition

Imaging type(s)	Functional MRI (and structural MRI for coregistration).
Field strength	3T.
Sequence & imaging parameters	A magnetic (B0) field map was assessed to unwarp the functional images (TR=634ms, TE1=4.92ms, TE2=7.38ms, 40 slices, voxel size=2.9x2.9x3.0mm3, FOV=224mm). For the functional scans, T2*-weighted echo planar imaging sequences were used to obtain 2mm thick transversal slices (TR=2000ms, TE=30ms, flip angle=60∘, FOV=224). Additionally, a high-resolution T1 weighted anatomical image (TR=2500 ms, TE=2.12 ms, 256 slices, voxel size =0.8x0.8x0.9mm3) was collected for coregistration of the functional scans.
Area of acquisition	Whole-brain scan.

Diffusion MRI ☐ Used ☒ Not used

Preprocessing

Preprocessing software	Preprocessing was performed with SPM12. The images were first realigned and unwarped using the field maps, then coregistered to the structural image followed by a normalization to Montreal Neurological Institute (MNI) space. For the univariate analysis, the images were additionally smoothed with an 8mm full-width half-maximum Gaussian kernel. The multivariate analysis was applyed on unsmoothed data.
Normalization	Images were spatially normalized using SPM12's unified segmentation.
Normalization template	Data were normalized into standard stereotactic (MNI) space using SPM12's standard template (IXI549Space).
Noise and artifact removal	SPM12 realign and unwarp was used to correct for motion artifacts and geometric distortions.
Volume censoring	None.

Statistical modeling & inference

Model type and settings	On the first level, the functional MRI data was analyzed using general linear modeling (GLM) as implemented in SPM12. For the univariate analyses the GLM included one regressor per run and per emotion for the encoding task (6 regressors) and one regressor per emotion and stimulus category for the recognition task (8 regressors) as well as 6 run constants as regressors of no interest. The resulting 20 regressors were convolved with the canonical hemodynamic response function.

For this multivariate analysis, each individual trial of the encoding and recognition task was modelled as an individual regressor convolved with a hemodynamic response function along with six session-constants in one GLM per subject using SPM12. A high-pass filter of 128s was used to remove low-frequency drifts and serial correlations in the time series were accounted for using an autoregressive AR(1)-model.

Effect(s) tested

Group differences during memory testing: Flexible Factorial model with the between factors delay (1d vs. 28d), drug (Placebo vs. Yohimbine) and the within-factor picture-type (old vs. new).
Change from encoding to recognition: Flexible Factorial model with the between factors delay (1d vs. 28d), drug (Placebo vs. Yohimbine) and the within-factor task (recognition vs. encoding).
Multivariate memory reinstatement: Flexible Factorial model with the between factors delay (1d vs. 28d), drug (Placebo vs. Yohimbine) and the within-factor similarity (Encoding-Old-Similarity vs. Encoding-New-Similarity).

Specify type of analysis: ☐ Whole brain ☐ ROI-based ☒ Both

Anatomical location(s)

Anatomical locations were determined using probabilistic atlases.(Harvard-Oxford atlas) as well as using coordinates derived from a meta-analysis conducted on the neurosynth.org platform

Statistic type for inference
(See Eklund et al. 2016)

Voxel-wise.

Correction

FWE-correction aswell as Bonferroni Correction for the number of ROIs in each analysis.

Models & analysis

n/a	Involved in the study
☐	☒ Functional and/or effective connectivity
☒	☐ Graph analysis
☐	☒ Multivariate modeling or predictive analysis

Functional and/or effective connectivity

Psycho-Physiological interaction (PPI) analyses was applyed with SPM12. To this end, the first eigenvariate of the activity time course of the relevant ROI for old pictures and new pictures were extracted and included as seed in the PPI. A first-level model was set up including the seed, a vector coding the contrast of interest as well as an interaction term, computed as the element by element product of the first two regressors. The resulting interaction contrasts were then analyzed on the second-level by means of a flexible factorial model.

Multivariate modeling and predictive analysis

Representational Similarity Analysis (RSA) using a spherical searchlight approach was used to assess Encoding-Retrieval-Similarity (ERS). To increase the reliability by normalizing for noise, the beta-values resulting from the trial-wise GLMs (see above) were further transformed into t-statistics. Then a whole-brain-searchligt-analysis with a 3-voxel-radius sphere was centered on every voxel of the brain and subjected the resulting set of voxels to an RSA, i.e. calculating the similarity (Pearson's r) between pattern responses during the final run of the encoding task on experimental day 1 and during old items in the recognition task on day 2 (Encoding-Old-Similarity, EOS) and between pattern responses during the final run of encoding and the corresponding (matched by visual complexity, valence, occurrence of humans, animals or objects) new items of the recognition task (Encoding-New-Similarity, ENS). The resulting r-maps were further Fisher z-transformed.
